# An Account of Two Cases of Amenorrhœa, with Some Observations on the Use of the Root of Madder in That Disease

**Published:** 1790

**Authors:** Peter Copland

**Affiliations:** Surgeon at Swayfield, near Colsterworth, in Lincolnshire


III. *An Account of two Cases of Amenorrhœa, with some Observations on the Use of the Root of Madder in that Disease. Communicated in a Letter to Dr.* Simmons *by Mr.* Peter Copland, *Surgeon at Swayfield, near Colsterworth, in Lincolnshire.*

SOME time in January, 1785, I was desired to see Anne Musson, of Woolsthorpe, aged sixteen years, of a thin habit, but florid complexion. For some months she had been troubled, at three weeks and a month's distance, with a sense of weight at her stomach, gradually increasing, accompanied with nausea, which, in a few days, always amounted to a vomiting of blood, followed by a perfect relief of the affection of her stomach. The menses had not yet appeared.

A benevolent person, who occasionally administered assistance to her poor neighbours, and who pointed out this patient to me, requested that I would order such medicines as might be in her power to prepare. Accordingly an infusion of bitters, with iron filings, in new ale, was directed to be taken in the quantity of half a pint three times a day. This she continued till

March

March 17th without any advantage; at which time half a drachm of the root of madder, in powder, was given in each dofe of the medicated ale. When fhe had nearly taken an ounce of the root, a coloured difcharge, in fmall quantity, took place from the vagina, and, the powder having been continued to the amount of an ounce from the beginning, at the fucceeding period a complete menftrual difcharge occurred.

In May and June following no periodic appearance taking place, yet without the former affection of the ftomach recurring, I advifed, July 6th, the powder of madder root to be taken in the fame quantity as before in a little of her common drink. Before an ounce was ufed the difcharge took place, and continued to return without any irregularity. She has fince married, and has lately lain in of her firft child.

As at the application of the above patient relief was principally requefted for the affection of her ftomach; as fhe, with her mother, have repeatedly affirmed the certainty of the cafe without any temptation to deceive; as cafes of epiftaxis, &c. vicarious to the menftrual difcharge, charge, are recorded by Dr. Hamilton *, and as hæmatemeſis is allowed to have happened, (but the inſtances are mentioned as very uncommon) from the retention of the menſes, by Dr. Cullen †, I think there can be little doubt of the above as a fact. If we add to the ſymptoms of ſenſe of weight at the ſtomach, nauſea, and vomiting, the conſideration that no cough at any time attended, the hæmorrhage muſt have evidently proceeded from the ſtomach.

Although from the account of the preceding caſe the emmenagogue effect of the root of madder is repeatedly ſeen, yet in other inſtances it hath repeatedly failed.

In a caſe of retained menſes ſimply, accompanied with a pale countenance, difficult breathing upon motion, œdematous ſwelling of the feet and ancles, and general debility, the root of madder taken in the quantity of an ounce in half-drachm doſes three times a day had not the wiſhed effect; and *tinctura ferri muriati* taken in drops in half a pint of cold chamomile tea three times a day, to the amount of an ounce,

* Dr. Duncan's Medical Commentaries, Vol. I. dec. 2.

† Firſt Lines of the Practice of Phyſic, 1786, Vol. III. page 53.

had had no other effect than mending the general health: the discharge, however, appeared a few months after, no additional means having been used.

Another case occurred in which the person had reached her twenty-seventh year without having experienced the usual periodic discharge, and was in every respect in perfect health; but having heard of the above cases, she was anxious to make use of the same means with the view of inducing the menstrual flux. The root of madder was, therefore, given in half-drachm doses three times a day, to the amount of two ounces, but without any effect whatever. I lately understood from her that she still continues without any appearance of the menses; that she never had either leucorrhœa or piles; that some years ago, upon taking medicines from a neighbouring practitioner, she perceived a small stain on her linen; that she feels herself indisposed monthly; and that she is now in her thirty-first year. At the same time she had no inclination again to try the effect of any medicine.

I have thus taken the liberty, Sir, of offering to you, with two unfrequent cases of amenorrhœa, an account of the emmenagogue property of the root of madder as it hath occurred to me, with the view of ſoliciting the experience of others, who may have had more opportunities of its uſe in the treatment of a diſeaſe which is but too often the ſource of much anxiety to the amiable part of ſociety.

*Swayfield,*
July 8, 1790.

